# Wastewater-based surveillance and early warning–forecasting framework for norovirus: a two-year longitudinal study in Shenzhen, China

**DOI:** 10.3389/fmicb.2026.1821841

**Published:** 2026-05-12

**Authors:** Shiju Chen, Hailong Zhang, Jia Wan, Yinghui Li, Xianghui Shi, Haiduan Lin, Rongling Pan, Sha Zhou, Chuande Chen, Cuiping Kuang

**Affiliations:** 1 Luohu Center for Disease Control and Prevention, Shenzhen, Guangdong, China; 2 Shenzhen Center for Disease Control and Prevention, Shenzhen, Guangdong, China; 3 Futian Center for Disease Control and Prevention, Shenzhen, Guangdong, China; 4 Longgang Center for Disease Control and Prevention, Shenzhen, Guangdong, China; 5 School of Public Health, Guangdong Medical University, Dongguan, Guangdong, China; 6 School of Public Health, University of South China, Hengyang, Hunan, China

**Keywords:** early warning, infectious disease surveillance, moving epidemic method, norovirus, Poisson regression model, wastewater-based epidemiology

## Abstract

**Objective:**

Norovirus (NoV) is a leading cause of acute gastroenteritis globally; however, traditional clinical surveillance underestimates its true infection burden. Wastewater-based epidemiology (WBE) offers a novel approach for comprehensive viral monitoring. This study aimed to develop and validate a practical WBE framework integrating a two-tiered early warning system and trend forecasting to support public health interventions against NoV.

**Methods:**

A two-year (August 2022–August 2024) WBE study was conducted in Shenzhen. NoV in influent wastewater samples from five wastewater treatment plants was monitored using reverse transcription–quantitative polymerase chain reaction (RT-qPCR). We employed a clinical data-calibrated approach to derive estimates of NoV cases from reported infectious diarrhea data; these estimates served as the gold standard. Using this gold standard, we employed the Moving Epidemic Method (MEM) to establish and validate a two-tiered early warning system based on wastewater NoV concentrations. In addition, we developed a Poisson regression model (PRM) to forecast NoV infection trends.

**Results:**

Wastewater NoV loads exhibited distinct seasonal fluctuations. The clinical data-calibrated estimates correlated strongly with wastewater viral concentrations (*r* = 0.75, *p* < 0.01). The MEM-based scheme achieved 100% detection of early stage moderate-level epidemics (sensitivity = 89.6%, specificity = 79.3%) and a 60.0% early warning rate for high-level epidemics, with a negative predictive value of 95.2%. Additionally, the PRM enabled one-week-ahead forecasts of NoV infection trends.

**Conclusion:**

WBE monitoring effectively captures the seasonal fluctuations of NoV infections in the population. This study provides a practical WBE framework integrating a two-tiered early warning system with one-week-ahead trend forecasting, thereby enabling the transformation of passive monitoring into an actionable public health tool for NoV.

## 1 Introduction

Norovirus (NoV) remains the leading cause of sporadic and epidemic acute gastroenteritis (Ahmed et al., 2014) and consists of two major epidemic subtypes: NoV genogroup I (GI) and genogroup II (GII) (Li et al., 2023). With notably high viral activity during winter and spring (Chen et al., 2023), it is also characterized by multi-channel (e.g., contaminated food and water, aerosols, and direct contact) transmission (Tan et al., 2024). Its prevalence is further complicated by a relatively high proportion of asymptomatic carriers (Shah and Hall, 2018). The traditional case reporting mechanism, serving as a passive monitoring scheme, cannot provide sufficient data for early warning of NoV prevalence, thereby limiting the ability to accurately reveal the actual epidemic situation.

Wastewater-based epidemiology (WBE) has emerged as a powerful monitoring tool in response to deficiencies in traditional reporting mechanisms (Verani et al., 2024). During the COVID-19 pandemic, WBE has proven to be efficient in tracking virus trends, leading to the launch and implementation of initiatives such as the National Wastewater Monitoring System in the United States (Centers for Disease Control and Prevention, 2022), the National Wastewater Monitoring Program in the Netherlands (van Boven et al., 2023), and the Integrated Wastewater Monitoring System in Hong Kong (Ng et al., 2023). WBE has been verified to be feasible for the early detection of bacterial, viral, and parasitic diseases (Carmo Dos Santos et al., 2024; Hassard et al., 2023; Ofori et al., 2024). More importantly, WBE can be applied to monitor NoV (Guo et al., 2022), given its fecal–oral transmission route, thereby serving as a non-invasive, population-level method for monitoring the NoV prevalence.

According to existing studies, the concentration of NoV in wastewater has a significant linear correlation with the number of NoV cases in the population, the number of infectious diarrhea cases, and the number of population outbreaks (Huang et al., 2022; Hotta et al., 2024; Boehm et al., 2024; Lu et al., 2021; Yu et al., 2024; Walker et al., 2024). Meanwhile, physical and chemical parameters (e.g., pH and temperature) may be key factors influencing the presence of viruses (Anneser et al., 2022; Krivoňáková et al., 2021; Rodríguez Rasero et al., 2022; Plaza-Garrido et al., 2023), highlighting the significance of active epidemiological monitoring of NoV via wastewater. However, a majority of the current studies were conducted mainly to clarify the correlation between viral concentration and population infection, with the absence of an integrated early warning and forecasting framework based on wastewater monitoring data. As a result, WBE has not been fully developed into a practical decision-support tool for public health interventions against NoV.

The Moving Epidemic Method (MEM), as a tool for establishing epidemic thresholds, has been widely used in the surveillance of respiratory diseases such as influenza. Its core advantage lies in distinguishing epidemic and non-epidemic periods using historical data and calculating accurate early warning thresholds with confidence intervals, which can adapt well to different epidemic characteristics and provide clear quantitative criteria for public health interventions (Jiang et al., 2022; Khaleel et al., 2024). For predictive modeling, the Poisson regression model (PRM) performs well in trend forecasting with limited datasets and is particularly suitable for fitting and projecting count data such as infection cases (Xu et al., 2025). Collectively, MEM holds potential for the development of graded early warning thresholds and PRM for the construction of a short-term infection prediction framework.

Therefore, supported by 2 years of continuous monitoring at five wastewater treatment plants (WWTPs) in Shenzhen, this study aimed to develop and validate early warning thresholds and prediction models for NoV. Given the large gap between reported and actual NoV cases, we estimated the true scale of NoV infections by combining clinical surveillance data, thereby providing core data to support the subsequent development of a warning and prediction framework.

## 2 Methods

### 2.1 Site selection

Sampling in this study was completed at five WWTPs in Shenzhen. Among these, four WWTPs are located in densely populated areas (e.g., Luohu District and Futian District), serving a total population of 3.33 million. Another is located in the Yantian District, serving a population of 210,000, which facilitates the comparison of population density. The daily flow rate ranges from 40,500 to 261,500 m^3^/day.

### 2.2 Sample collection

Sampling in our study was conducted once a week from 18 August 2022 to 23 August 2024. A 24-h quantitative sampling was adopted, given the daily variations in wastewater composition. In brief, wastewater samples (after grid and gravel chamber treatment) were collected, starting at 9 a.m. every 2 h, with 400 mL collected each time and stored at 4 °C. An AWRS SAMPLER (Model: 9503700, HACH Company) was used for sampling. At 9:10 a.m. the next day, 500 mL of the mixed sample was collected and transported under refrigerated conditions to the laboratory. To ensure laboratory safety, all wastewater samples were subjected to thermal inactivation at 56 °C for 30 min prior to further processing.

### 2.3 Sample concentration

Viral particles from the wastewater samples were concentrated via modified polyethylene glycol (PEG) precipitation (Zheng et al., 2023).

For pre-centrifugation, 45 mL of the wastewater sample was centrifuged at 2,000 g for 2 min after transferring it into a 50 mL centrifuge tube. After collection of the supernatant, the remaining wastewater solid fraction was discarded, and the supernatant was stored at 4 °C for subsequent use.

For PEG precipitation, 40 mL of the pre-centrifuged supernatant was added to a 50 mL centrifuge tube containing 4 g of molecular biology-grade PEG 8000 (10% w/v) and 0.8 g of NaCl (2% w/v). The mixture was shaken until complete dissolution of PEG for further incubation at 25 °C and 180 rpm for 2 h. The incubated mixture was then centrifuged at 4,750 × g for 30 min to obtain the resulting 2 mL precipitate (with the supernatant discarded) and transferred to a new tube.

For the final concentration, 2 mL of the precipitate was centrifuged at 20,000 × g for 2 min. The precipitate was resuspended in 500 μL of protective solution after removal of the supernatant and then stored at −80 °C for further use (Plaza-Garrido et al., 2023).

### 2.4 Viral RNA extraction and quantification

The extraction of viral RNA from 200 μL of the concentrated sample was performed using the automatic nucleic acid extraction system EXM 6000 (ZYBIO, Chongqing, China) and the Nucleic Acid Extraction Kit based on magnetic bead technology (ZYBIO, Chongqing, China). The final elution volume was 50 μL. Quantification was performed using the NoV GI/GII Nucleic Acid Detection Kit (MEIZHENG BIO-TECH, Shandong, China), which included a standard DNA template. The standard DNA was serially diluted tenfold to generate a standard curve (range: 10^6^–10^1^ copies/μL), serving as the positive control to verify the efficiency of polymerase chain reaction (PCR) amplification. In each PCR run, a negative control (nuclease-free water) was used to monitor contamination. Real-time PCR was performed using an Applied Biosystems 7500 Real-Time PCR System. The genome copies of NoV GI and GII in the concentrated samples were calculated based on the standard curve. The results are expressed as genome copies per liter (gc/L) of wastewater.

For samples with negative NoV (GI/GII) detection (i.e., viral load below the limit of detection), linear interpolation was used to impute the missing values. In brief, the estimation of the viral load of the missing sample was completed based on the linear regression of valid viral load data from the nearest preceding and subsequent samples, which could ensure continuous temporal concentration curves while avoiding overestimated or underestimated epidemic trends. Meanwhile, linear trend interpolation was applied only to occasionally negative samples during continuous monitoring. The interpolated values must not differ by more than 10-fold from the concentrations of the two nearby weeks to avoid inaccuracies.

### 2.5 Population infection data

For data acquisition, the China Disease Prevention and Control Information System^1^ was used to directly retrieve information on NoV infection cases (catchment-NoV cases), infectious diarrhea cases (catchment-diarrhea cases) within the WWTP catchment, and citywide NoV infection cases (citywide NoV cases). Notably, owing to privacy and data security protocols, this system is restricted to authorized access (for authorized public health institutions and researchers) only. The filtration of cases was completed based on the patients’ current addresses, onset dates, and report dates. Given the incorporation of only anonymized and aggregated surveillance data (without individual identifiers) in our study, ethical review and approval were waived according to the Chinese research ethics guidelines for public health surveillance data.

### 2.6 Estimation of infection numbers and establishment of predictive models

#### 2.6.1 Estimation of NoV infections based on clinical data calibration

First, the positive rates of NoV were obtained from the NoV detection results of infectious diarrhea samples from sentinel hospitals monitored across the city during the same period. Data were sourced from the Public Health Information Platform of the Shenzhen Center for Disease Control and Prevention (Shenzhen CDC^2^). Similarly, this platform is restricted to authorized access owing to privacy protection and data security protocols.

Then, the positive rate was smoothed by adopting a two-week moving average (encompassing the 2 weeks before and after the corresponding time points) combined with sample size calibration. Specifically, the product of the positive rate and sample size for each week within the five-week window was summed to compute the total number of positive cases. Subsequently, the calibrated positive rate was derived by dividing the total positive case count by the sum of the sample sizes across the 5 weeks.

Next, the estimated number of NoV cases (estimated NoV cases) was calculated. Considering the regional medical treatment characteristics and a previous study in the United States (Schmidt et al., 2022), a uniform correction parameter of 20% was used as the medical-seeking rate for infectious diarrhea. The estimated NoV cases were then computed by dividing the number of catchment-diarrhea cases by the rate of seeking medical treatment for infectious diarrhea and then multiplying by the NoV-positive rate.

#### 2.6.2 Early warning thresholds for NoV infections based on MEM

The baseline dataset was the estimated clinical data-calibrated NoV GII cases from week 34 of 2018 to week 33 of 2022. The mean (μ) and standard deviation (SD) of this dataset were calculated to define the gold standard for epidemic status. Specifically, moderate- and high-level epidemics were defined as weekly estimated cases exceeding μ and μ + 2 SD, respectively.

Subsequently, this study constructed warning thresholds using the MEM, with the window size, step size, and percentile included as the core parameters. The step size was set to half the value of the window size for each candidate combination. Regarding the ranges of candidate parameters, the window size was 4/6/8/10/12/16/20/24 weeks, and the percentile was defined as 50%/75%/85%/90%/92.5%/95%/97.5%/99.5%. All candidate parameter combinations were traversed with the F1 score as the core selection index. The combination with the highest F1 score was determined as the optimal model parameter for each epidemic status.

Finally, multiple indicators were incorporated to assess the early warning effectiveness after the establishment of the warning threshold. The specific evaluation indicators included sensitivity, specificity, positive predictive value, negative predictive value, F1 score, and early warning rate.

#### 2.6.3 Predictive models for NoV-related infections

In this study, the estimated NoV case numbers from week 34 of 2018 to week 33 of 2022 were used as reference values. A PRM was developed to forecast NoV infection trends and the number of cases. The independent variable was the flow rate–weighted average viral load (FWAV) of NoV from the five WWTPs. This variable was calculated on a weekly basis, followed by base-10 logarithmic transformation (log-transformation).

Environmental and wastewater parameters, including temperature, flow rate, pH, chemical oxygen demand (COD), and ammonia nitrogen, are critical factors regulating NoV survival, adsorption, and detectable concentrations in wastewater (Plaza-Garrido et al., 2023). Their inclusion in the model allowed us to account for potential confounding effects and improve model robustness (Shi et al., 2025). Weekly average temperatures were retrieved from the China Meteorological Administration website.^3^ The total volume of wastewater treated on each sampling day across the five WWTPs, as well as the mean concentrations of ammonia nitrogen (NH_3_-N), pH, COD, and biological oxygen demand (BOD) in the influent wastewater, were provided by the respective WWTPs.

Data preprocessing involved the normalization of the raw independent variables, with the creation of lagged features. The dependent variable was the clinical data-calibrated estimated NoV cases. To enhance the model performance, the fitting results were compared with retrospectively accumulated case counts over 3-day, 5-day, 7-day, 10-day, and 14-day periods as the dependent variables. The simulation differences between scenarios without lag and those with a 7-day lag were assessed simultaneously. The case counts were based on the report date to meet the requirements of practical applications.

In addition, sampling in our study was conducted once a week for 106 weeks. Critically, the construction of the prediction models was realized using data from the first 77 weeks as the training set, and the prediction accuracy was evaluated with data from the remaining 29 weeks as the test set. Eventually, the adjusted R-squared (R^2^) and root-mean-square error (RMSE) were utilized for assessing the performance of the constructed models.

### 2.7 Statistical analysis

All data were entered into Microsoft Office Excel 2019. The FWAV, which represents the overall viral concentration across all sampled WWTPs, was calculated by summing the product of the viral concentration and daily flow rate at each plant and dividing by the total daily flow across all WWTPs. Viral concentrations across the four seasons were compared using the Wilcoxon signed-rank test. R (version 4.4.2; R Core Team, 2024) was used for data visualization. The FWAV values were log-transformed prior to Spearman’s rank correlation analysis, early warning implementation, and predictive modeling. The MEM-based early warning threshold setting and construction of the PRM predictive model were implemented using Python 3.12.7. In this process, generative artificial intelligence (GenAI) tools were used to assist in generating the initial code frameworks for MEM algorithm implementation, model construction, and parameter tuning. All generated codes were further reviewed, validated, and modified to ensure analytical accuracy, methodological validity, and alignment with the study objectives. A significance level of 0.05 was adopted for all statistical tests.

## 3 Results

### 3.1 Detection rate and viral load of NoV

Validation by RT-qPCR showed that the amplification efficiencies of GII and GI ranged from 101.59 to 113.64% and from 96.36 to 113.61%, respectively, with all *R*^2^ values > 0.9947. These results were consistent with the acceptable range for qPCR-based quantification, confirming the reliability of the quantitative results.

A total of 518 wastewater samples were collected from 18 August 2022 to 23 August 2024. The overall detection rates of NoV GI and GII were 91.31% (473/518) and 94.98% (492/518), respectively. Among these samples, 463 (89.38%, 463/518) tested positive for both NoV GI and GII. NoV was extensively distributed in the raw wastewater samples from the five WWTPs. The detection rates for NoV GI and GII ranged from 89.00 to 94.23% and 93.00 to 97.12%, respectively (Table 1).

**Table 1:** Detection of NoV (GI and GII) in five WWTPs in Shenzhen from August 2022 to August 2024.

Wastewater treatment plants	Number of samples	NoV GI		NoV GII	
		Positive (*n*)	Detection rate (%)	Positive (*n*)	Detection rate (%)
BH	100	89	89.00	93	93.00
BJ	106	96	90.57	102	96.23
HH	104	95	91.35	96	92.31
LF	104	98	94.23	100	96.15
YT	104	95	91.35	101	97.12
Total	518	473	91.31	492	94.98

**Figure 1:**
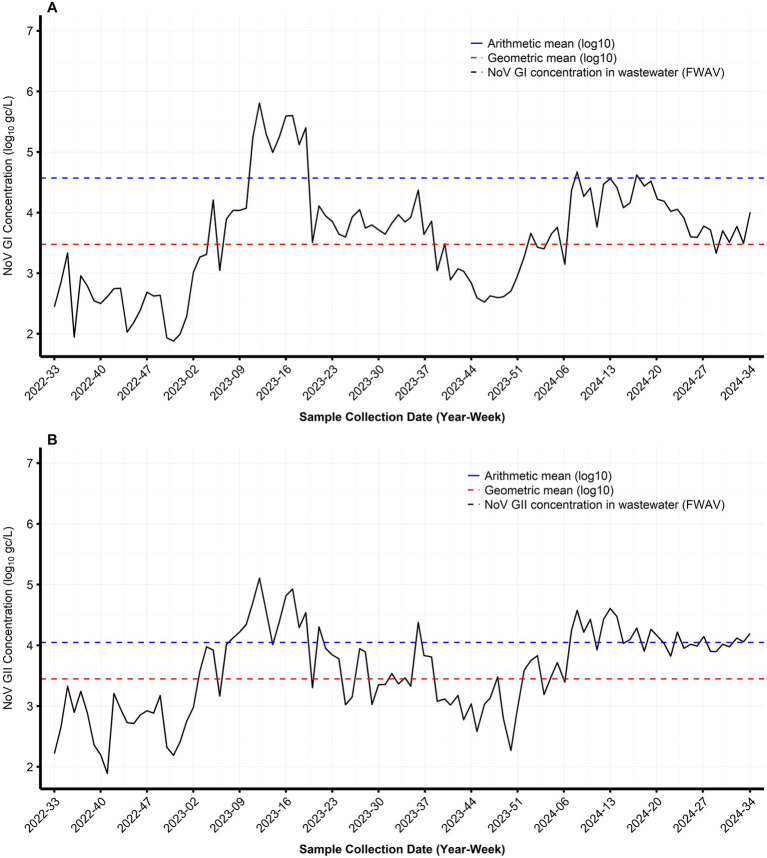
Temporal dynamics of NoV concentrations in wastewater from five WWTPs in Shenzhen (2022–2024). **(A)** NoV GI concentration. **(B)** NoV GII concentration. For samples with negative NoV detection, missing values were imputed using the linear trend of the adjacent valid viral load data.

Among the valid samples detected (excluding interpolated data), the maximum NoV GI concentration across the five WWTPs was 2.06 × 10^6^gc/L, with an overall arithmetic and geometric mean values of 3.3 × 10^4^ gc/L and 3.67 × 10^3^ gc/L, respectively. For NoV GII, the maximum concentration across the studied plants was 3.22 × 10^5^ gc/L, with an overall arithmetic and geometric mean value of 1.1 × 10^4^ gc/L and 3.10 × 10^3^ gc/L, respectively. The temporal dynamics of the NoV concentrations in wastewater from the five WWTPs are shown in Figure 1.

### 3.2 Seasonal trends of NoV in wastewater

**Figure 2:**
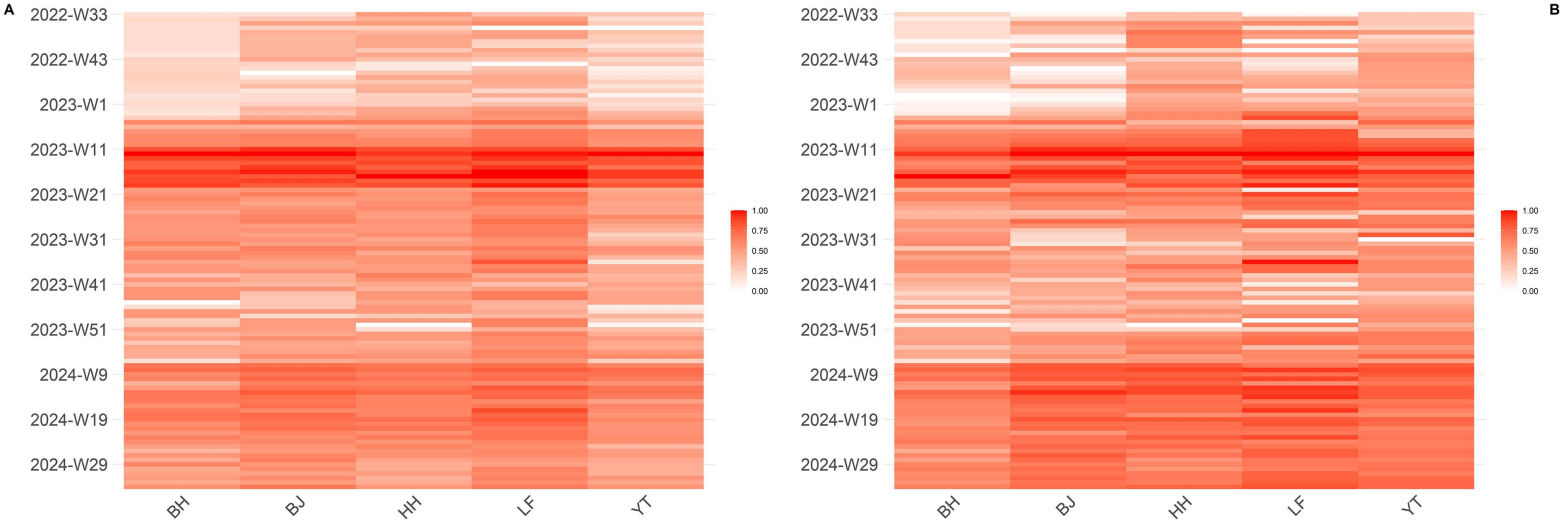
Heat map of the weekly concentrations of NoV in five WWTPs from August 2022 to August 2024 after log-transformation and min-max normalization. **(A)** NoV GI; **(B)** NoV GII.

In this study, year-round dynamic monitoring of NoV RNA concentrations was carried out across five WWTPs from the summer of 2022 to that of 2024. Distinct seasonal fluctuations were observed in the concentration of NoV in the wastewater. Concentration peaks were observed primarily between weeks 11 and 20 in 2023 and between weeks 9 and 19 in 2024. Additionally, the highest peak occurred at week 12 of 2023, with the 2023 peak being more pronounced than that of 2024 (Figure 2).

**Figure 3:**
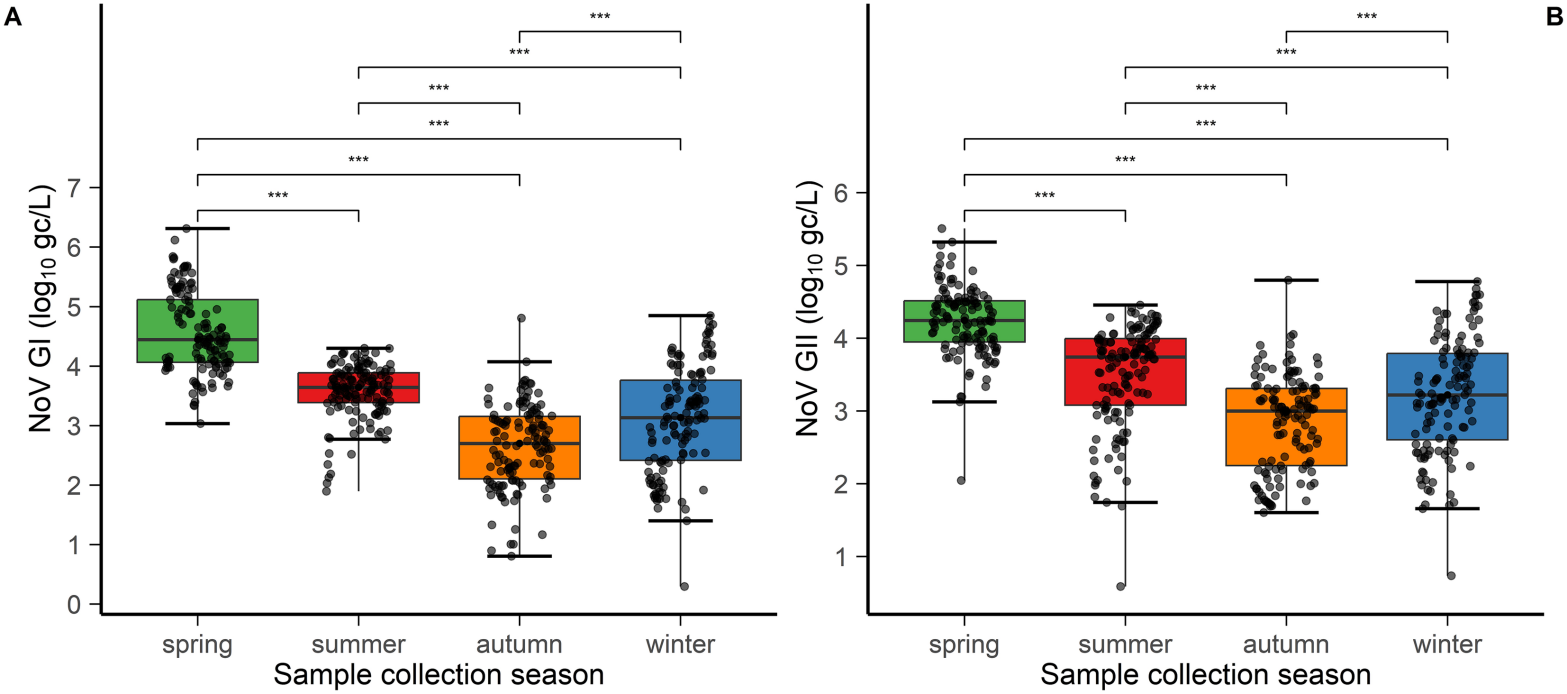
Box plot of NoV concentrations in the four seasons in the five WWTPs. **(A)** NoV GI concentrations; **(B)** NoV GII concentrations. Wilcoxon signed-rank test: *p < 0.05, **p < 0.01, and ***p < 0.001, with statistically significant differences. NS, No statistical difference. Seasonal division: Spring = March–May, summer = June–August, autumn = September–November, and winter = December–February.

Consistent with these temporal patterns, spring was the season with the highest viral load for both NoV GI and GII, followed by winter. As shown in Figure 3, for both genotypes, the concentrations in winter and spring were significantly higher than those in summer and autumn (*p* < 0.05).

### 3.3 Relationship between NoV concentration in wastewater and the number of NoV cases

#### 3.3.1 Characteristics of clinical case data and of predominant genotypes

From week 33 of 2022 to week 34 of 2024, 1,029 NoV infection cases and 24,888 infectious diarrhea cases were reported within the service areas of the five WWTPs. These two datasets exhibited significant correlation, with a Spearman correlation coefficient of 0.56 (*p* < 0.01). Additionally, 7,565 citywide NoV cases were reported during the same period. Meanwhile, the trend of the citywide NoV cases was consistent with that of the catchment-NoV cases, with a Spearman correlation coefficient of 0.76 (*p* < 0.01).

From 2022 to 2024, 375 NoV-positive samples were identified from fecal specimens collected from patients suspected of having viral diarrhea via the Public Health Information Platform of Shenzhen CDC. Among these, 83.2% were identified as NoV GII (312/375), 10.7% as NoV GI (40/375), and 6.1% as mixed infections (23/375). Therefore, NoV GII is the predominant genotype that infects the population in Shenzhen. Thus, our subsequent analyses focused on the correlation between the NoV GII RNA load in the five WWTPs and the number of reported cases.

#### 3.3.2 Correlation between wastewater NoV GII concentrations and clinical case counts

The log-transformed FWAV of NoV GII exhibited a correlation with the catchment-diarrhea cases, showing a Spearman correlation coefficient of up to 0.66 (Table 2). Moreover, this correlation was notably stronger than that with catchment and citywide NoV cases.

**Table 2:** Spearman correlation coefficients between log-transformed FWAV of NoV GII and prior clinical case counts.

Prior case accumulation window	Catchment-NoV cases	Citywide NoV cases	Catchment-diarrhea cases	Estimated NoV cases
3-day prior cases	0.32	0.36	0.62	0.74
5-day prior cases	0.35	0.37	0.63	0.74
7-day prior cases	0.30	0.38	0.64	0.74
10-day prior cases	0.35	0.37	0.64	0.75
14-day prior cases	0.35	0.36	0.66	0.75

All correlation coefficients were statistically significant (all *P* < 0.05). The number of cases was counted based on the onset date.

### 3.4 Results of NoV infection number estimation

The total estimated number of NoV infections reached 27,993 (95%CI: 24,507–36,598) between week 34 of 2018 and week 34 of 2024. Further counting by monitoring weeks revealed a fluctuation of the estimated cases across years: 1,224 cases in 2018 (starting from week 34, 95%CI: 957–1,498), 6,326 cases in 2019 (full year, 95%CI: 5,186–7,508), 3,596 cases in 2020 (95%CI: 2,889–4,429), 5,772 cases in 2021 (95%CI: 4,921–7,043), 912 cases in 2022 (95%CI: 667–1,332), 5,154 cases in 2023 (95%CI: 4,933–7,276), and 5,008 cases in 2024 (up to week 34, 95%CI: 4,952–7,512).

**Figure 4:**
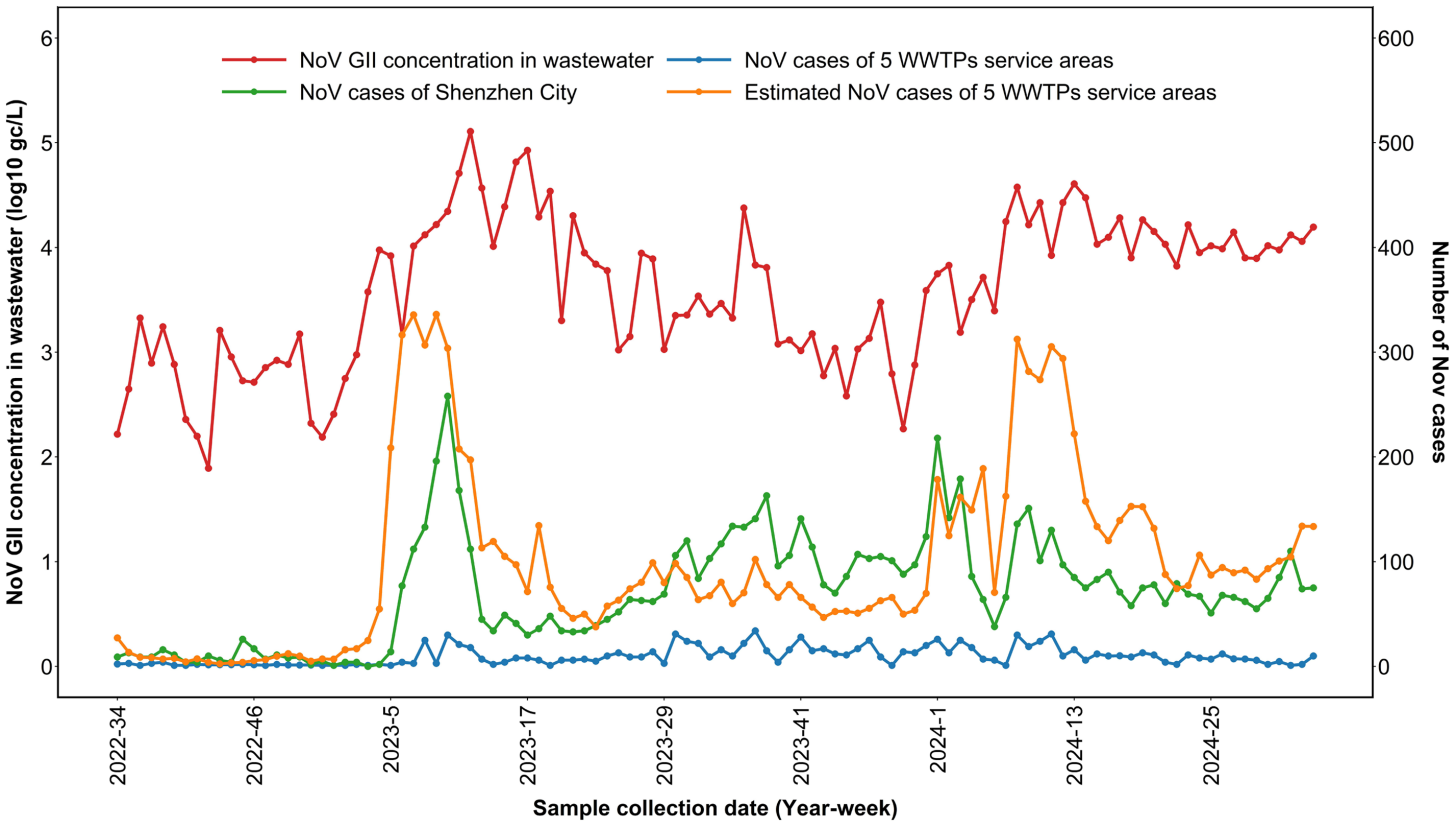
Temporal dynamics of wastewater NoV GII concentrations and different NoV case series.

Furthermore, distinct epidemic trends were observed in the estimated NoV cases, exhibiting two typical peaks: between weeks 5 and 13 of 2023 and between weeks 1 and 13 of 2024 (Figure 4). Noticeably, these two peak periods were closely aligned with the peak timings of citywide NoV cases (*r* = 0.62, *p* < 0.01) and catchment-NoV cases (*r* = 0.47, *p* < 0.01).

The estimated NoV cases (between weeks 33 of 2022 and weeks 34 of 2024) correlated strongly with wastewater NoV GII concentrations across all accumulation windows (Table 2), with the 10-day prior-case window yielding the highest correlation coefficient (Spearman’s *r* = 0.75, *p* < 0.01). In particular, this correlation was significantly higher than that between catchment NoV cases and these viral concentrations (*r* = 0.35, *p* < 0.05) and that between catchment infectious diarrhea cases and the concentrations (*r* = 0.64, *p* < 0.01).

Notably, in February 2023, citywide NoV infections surged significantly and were effectively captured by wastewater viral concentrations, showing a distinct synchronous peak during this period. Both indicators remained low, with consistent trends prior to this surge (August 2022–January 2023).

### 3.5 Early warning thresholds

Early warning lines for NoV wastewater monitoring were established in our study using MEM. Table 3 summarizes the optimal parameter configuration and threshold for early warning. Specifically, the warning threshold corresponding to moderate-level epidemics was 83 estimated NoV infection cases and 4200.05 gc/L of NoV concentration in wastewater. Simultaneously, for high-level epidemics, the thresholds were 232 estimated NoV infection cases and 16030.47 gc/L of NoV concentration in the wastewater.

**Table 3:** Optimal parameter configuration and threshold for early warning.

Warning level	Window size (weeks)	Step size (weeks)	Percentile (%)	Original threshold (gc/L)	Log threshold (log10 gc/L)
Moderate-level	6	4	50	4200.05	3.62
High-level	8	4	97.5	16030.47	4.20

**Figure 5:**
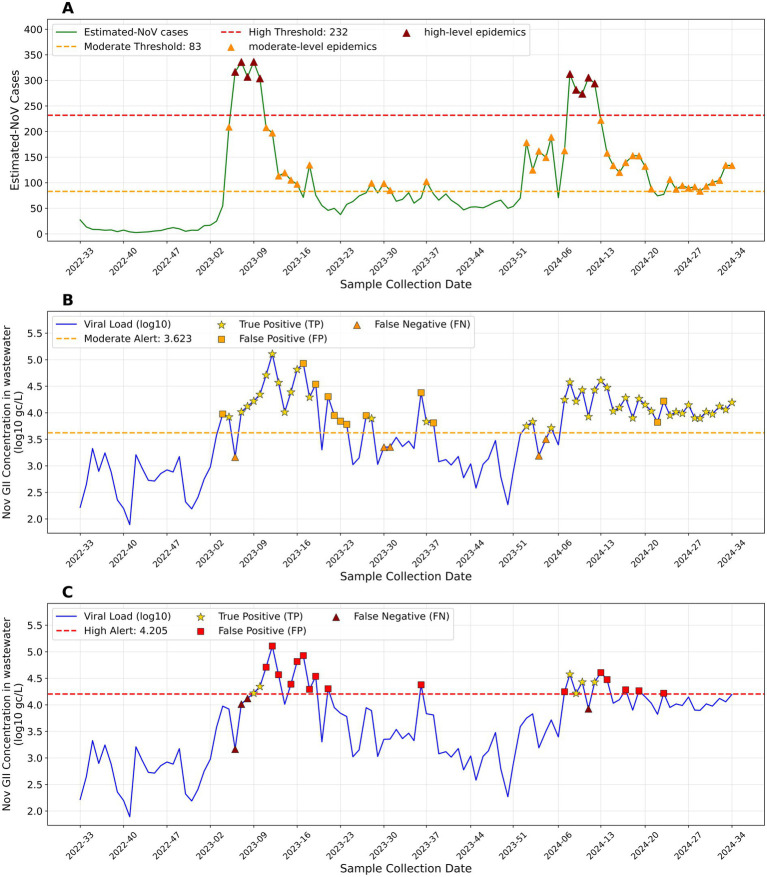
Visualization of NoV wastewater monitoring alert thresholds and alert performance. **(A)** Time series of estimated NoV cases with alert thresholds. **(B)** Moderate-level epidemic alert performance. **(C)** High-level epidemic alert performance.

Regarding the performance of the early warning line, the moderate epidemic warning threshold had a sensitivity of 89.6%. The early warning rate was 100.0%. It also maintained a relatively high specificity of 79.3%, a positive predictive value of 78.2%, and a negative predictive value of 90.2%. Correspondingly, the wastewater NoV GII viral load (log10) (Figure 5B) exceeded the moderate alert threshold during periods of elevated estimated NoV cases (Figure 5A), with most alerts corresponding to true-positive signals.

Meanwhile, the high-level epidemic warning system possessed high specificity (83.3%) and negative predictive value (95.2%). Despite its relatively conservative sensitivity (60.0%), it had an early warning rate of 60.0%. As depicted in Figure 5C, the high alert threshold effectively captured the two major peaks of the estimated NoV cases.

The performance indicators of the early warning lines are listed in Table 4.

**Table 4:** Performance of early warning lines.

Performance indicators	Moderate-level	High-level
Sensitivity	89.6%	60.0%
Specificity	79.3%	83.3%
Positive predictive value	78.2%	27.3%
Negative predictive value	90.2%	95.2%
F1 score	0.84	0.38
Early warning rate	100.0%	60.0%

### 3.6 Development of predictive models

As indicated in Table 5, the performance of the prediction model was assessed to evaluate the wastewater data-based early warning capability. Among all combinations of models, lag settings, and cumulative-case time frames, the simulations yielded optimal results when using the PRM with a 7-day lag setting, where the 10-day prior cumulative number of cases served as the dependent variable. This combination demonstrated a superior model fit compared to the other combinations, as evidenced by the highest *R*^2^ value and relatively low RMSE (*R*^2^ = 0.67, RMSE = 62.74). Thus, based on the optimal performance of PRM, wastewater monitoring data can enable one-week-ahead early warning of NoV infections.

**Table 5:** Model fit statistics for wastewater NoV GII concentrations to estimated NoV cases.

Lag setting and dependent variable	*R* ^2^	RMSE
No lag		
3-day prior cases	0.47	24.33
5-day prior cases	0.51	41.15
7-day prior cases	0.54	53.71
10-day prior cases	0.60	70.19
14-day prior cases	0.59	97.86
7-day lag		
3-day prior cases	0.54	22.42
5-day prior cases	0.59	37.18
7-day prior cases	0.62	48.43
10-day prior cases	0.67	62.74
14-day prior cases	0.67	86.60

**Figure 6:**
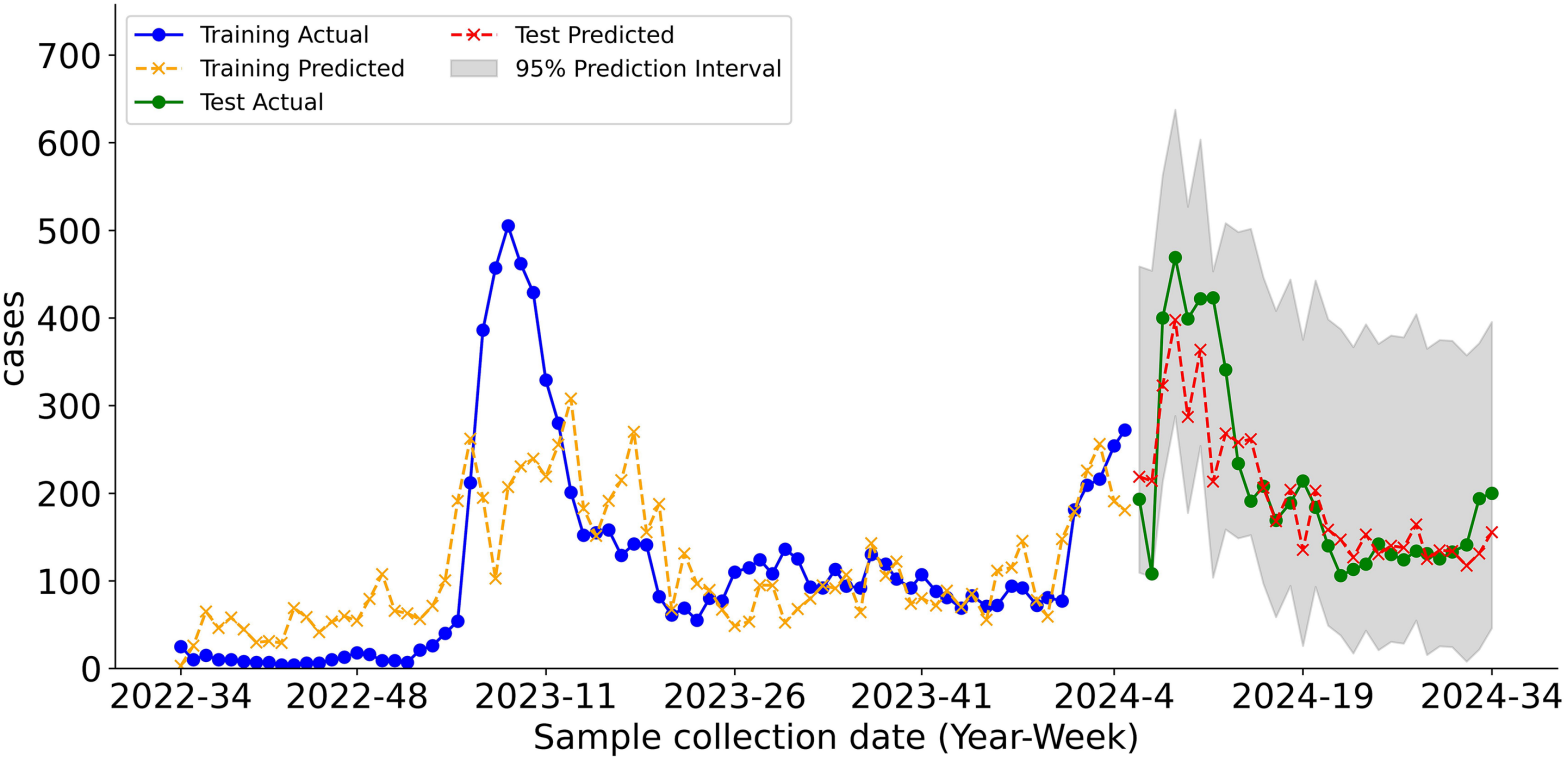
Model simulation and prediction results of the PRM for 10-day prior cases with a 7-day lag, showing a comparison between the actual and predicted values in the training and test datasets.

Figure 6 depicts the time-series comparison between the predicted and actual values (i.e., the estimated NoV cases) of the PRM on the test set. The regression results of the PRM (Table 6) further verified that FWAV was the most significant covariate for prediction, and wastewater flow, average temperature, pH, COD, BOD, and NH₃-N were identified as statistically important factors.

**Table 6:** Model results of the PRM.

Variable	Coefficient	Standard error	*z* value	*p* value
FWAV	0.54	0.01	43.85	<0.001
pH	−0.38	0.03	−11.14	<0.001
Flow	−0.25	0.02	−11.96	<0.001
Average temperature	−0.23	0.02	−14.41	<0.001
COD	−0.21	0.02	−10.28	<0.001
BOD	0.20	0.02	8.49	<0.001
NH_3_-N	−0.11	0.02	−5.33	<0.001

## 4 Discussion

To establish a surveillance and early warning framework for NoV prevalence in the population based on WBE, this study relied on 2 years of continuous monitoring data from five WWTPs in Shenzhen. By innovatively integrating clinical data to calibrate and estimate the number of NoV infections in the population, this study verified the correlation between wastewater NoV concentrations and the actual population infection burden. Additionally, graded early warning thresholds for wastewater NoV were established using MEM and a short-term prediction model for population NoV infections were developed in combination with PRM, providing a novel monitoring and early warning technical approach for NoV prevention and control in large, densely populated cities.

There were significant seasonal fluctuations in the concentrations of NoV GI and GII in wastewater, with higher levels in spring and winter, but lower levels in summer and autumn. Despite some differences in detection rates, these seasonal patterns are aligned with findings from other regions, such as Shenzhen and Hong Kong (Yue et al., 2024; Zheng et al., 2024). We also observed a marked change in NoV prevalence during the COVID-19 period. The calibrated NoV estimates declined sharply in 2022 and rebounded noticeably in early 2023, which is consistent with the relevant wastewater-based NoV monitoring results (Zheng et al., 2024; Bonanno Ferraro et al., 2025). This pattern indicates that strict population mobility restrictions effectively reduced NoV transmission, whereas relaxation of such measures led to a resurgence in viral spread.

During parameter optimization of the prediction model, we found that the model exhibited the best fit degree and prediction accuracy when a 10-day cumulative case window was adopted. This result was not accidental, and its core reason is likely closely related to the population shedding characteristics of NoV—this window duration is highly consistent with the previously reported median NoV shedding period of 11.5 days (Zheng et al., 2025), which exactly covers the key cycle of infected individuals from onset to peak viral shedding and then gradual decline. This window can capture the real dynamics of the population infection burden more comprehensively, thereby providing more reliable parameters for the model.

This study addresses a fundamental gap in NoV WBE by establishing a reliable “gold standard” to bridge wastewater signals and true community infection burden, thereby enabling the development of predictive tools. Based on epidemiological parameters within the wastewater service area, this study constructed an estimation formula for the true number of NoV infections for the first time. This newly developed formula overcomes the limitations of traditional estimation methods that rely solely on single clinically reported data through the integration of three key indicators: the number of infectious diarrhea cases in the service area, the NoV-positive rate in infectious diarrhea surveillance samples, and the medical consultation rate of infectious diarrhea individuals. The proposed estimation formula may facilitate the correction of the underestimation bias of clinically reported data, and the estimated values are closer to the true NoV infection level in the population.

As a methodological innovation, this study applied MEM to wastewater NoV monitoring. This method determines the optimal parameters through statistical analysis, providing a scientific quantitative basis for dividing epidemic intensity thresholds, which is more reasonable than the traditional method of determining thresholds based solely on the average levels or quartiles. Meanwhile, the combined application of MEM and PRM constructs a full-process system of “wastewater monitoring—graded early warning—short-term prediction,” providing a replicable new framework for the practical application of WBE in NoV prevention and control.

An early warning system based on wastewater monitoring supports graded and precise prevention and control for public health authorities. When wastewater NoV indicators reach the moderate-intensity threshold, basic interventions can be implemented promptly, including regional health education on hand hygiene and food safety, strengthened symptom monitoring in susceptible, crowded settings, and more frequent environmental cleaning and disinfection, allowing early intervention during epidemics. When a high-intensity threshold is triggered, public risk warnings can be issued, targeted training on epidemic response can be provided for key collective settings, the supply of high-risk foods can be suspended, group activities can be reduced in such places, and medical institutions can prepare corresponding personnel and supplies for potential increases in cases. The prediction system provides advanced forecasts of NoV epidemic trends and transmission intensity and supports resource allocation, policy development, and emergency response for public health departments. This framework shifts NoV prevention and control from passive response to active prediction, thereby improving the overall efficiency and accuracy of epidemic management.

Current WBE studies on NoV mainly focus on correlation analysis between changes in wastewater viral concentrations and the positive rate of population NoV monitoring (Malla et al., 2024; Maoz Atias et al., 2026; Markt et al., 2023). The main feature of this study is the correlation analysis between dynamic wastewater viral concentrations and clinically calibrated estimated infection numbers, which more directly reflects the corresponding relationship between wastewater signals and the actual population infection burden. Wastewater monitoring can serve as an important supplementary method for population clinical monitoring, with its core advantage being that it can reflect the dynamic changes of population infection status in real time (Guo et al., 2022) without being affected by factors such as individual medical-seeking behavior and lag in case reporting. The effectiveness of wastewater early warning is also closely related to the timeliness of wastewater sampling and the lag of population clinical visits. A concurrent WBE study on NoV achieved a one-month early warning for NoV epidemics (Yan et al., 2026), whereas the present study constructed a weekly forward-looking early warning model, consistent with the findings of Chacón et al. (2021). The difference in early warning times between the two essentially stems from the different population monitoring parameters and analysis scales adopted in these studies. The aforementioned study used monthly clinical monitoring positive rates as correlation parameters, whereas this study used weekly estimated infection numbers as core parameters, which were more refined in terms of temporal resolution and modeling granularity. The early warning designs were adapted to their respective research scenarios and analysis needs, providing differentiated references for the wastewater monitoring of NoV with different prevention and control cadences.

Several limitations of this study should be acknowledged when interpreting the results. First, as for sampling frequency and monitoring timeliness, existing NoV wastewater monitoring studies generally sample at a frequency of 1–2 times per week or month (Shrestha et al., 2024; Zheng et al., 2024; Yue et al., 2024; Hotta et al., 2024). However, this study, constrained by resources and costs, sampled per week, which, although comparable to most aforementioned studies, resulted in a lag in the wastewater monitoring results behind clinical case reporting. The sampling frequency should be appropriately increased when resources are permitted. Second, all wastewater samples in this study were subjected to thermal inactivation to ensure the biosafety of laboratory personnel, a measure that was particularly necessary when the project began in August 2022 during a period of strict COVID-19 prevention and control in China. Although this step effectively reduced the biohazard risks for the research team, it may have caused partial degradation of viral RNA and slightly reduced the sensitivity of NoV nucleic acid detection (Kevill et al., 2025). Third, there is a need to improve detection quality control systems. The PMMoV virus is usually selected as an internal reference or the MS2 phage as a process control virus in most relevant studies (Yue et al., 2024; Shrestha et al., 2024), and a study in Hong Kong, China, has systematically analyzed key quality parameters such as the limit of detection, limit of quantification, and RT-PCR inhibition effects (Zheng et al., 2024). However, this study lacked quality control measures. Future studies may refer to established methods to supplement duplicate parallel sample testing, inter-laboratory comparisons, and integration of internal controls to standardize the detection process and enable recovery rate correction. Finally, the key parameters used in this study to estimate NoV infection numbers were primarily obtained from the reference literature, which may deviate from the actual local conditions. Future specialized surveys should be conducted to characterize these key parameters to support the accurate estimation of the population-level infection scale.

## 5 Conclusion

The rise of the WBE has offered new insights into NoV monitoring, early warning, and prediction. In our study, wastewater NoV concentrations can suggest population infection fluctuations and share consistent seasonal trends according to 2 years of continuous monitoring at five WWTPs in Shenzhen. This study proposes a clinical data-calibrated approach that integrates infectious diarrhea cases, NoV-positive rates, and consultation rates, which yields promising results. Moreover, the estimated infections were highly correlated with NoV concentrations in wastewater. This study also establishes medium- and high-level early warning thresholds via the MEM based on these estimates and verifies that the PRM enables a one-week-ahead trend prediction. Overall, wastewater NoV monitoring serves as an effective supplementary surveillance tool to address the gap between underreported clinical cases and actual infections.

## Data Availability

The original contributions presented in the study are publicly available. This data can be found here: https://zenodo.org/records/19435132.

## Footnotes

1
https://10.249.6.18:8881/cdc/login

2
https://10.130.232.8:8085/szpcPlatform

3
https://www.cma.gov.cn/
